# A Scientometric-Analysis-Based Review of the Research Development on Geopolymers

**DOI:** 10.3390/polym14173676

**Published:** 2022-09-05

**Authors:** Kaffayatullah Khan, Waqas Ahmad, Muhammad Nasir Amin, Sohaib Nazar

**Affiliations:** 1 Department of Civil and Environmental Engineering, College of Engineering, King Faisal University, Al-Ahsa 31982, Saudi Arabia; 2 Department of Civil Engineering, COMSATS University Islamabad, Abbottabad 22060, Pakistan

**Keywords:** geopolymers, alternative binder, sustainable material, sustainable development, scientometric analysis

## Abstract

A scientometric-based assessment of the literature on geopolymers was conducted in this study to determine its critical aspects. Typical review studies are restricted in their capability to link disparate segments of the literature in a systematic and exact way. Knowledge mapping, co-citation, and co-occurrence are very difficult components of creative research. This study adopted an advanced strategy of data mining, data processing and analysis, visualization and presentation, and interpretation of the bibliographic data on geopolymers. The Scopus database was used to search for and retrieve the data needed to complete the study’s objectives. The relevant sources of publications, keyword assessment, productive authors based on publications and citations, top papers based on citations received, and areas actively engaged in the research of geopolymers are recognized during the data assessment. The VOSviewer (VOS: visualization of similarities) software application was employed to analyze the literature data comprising citation, bibliographic, abstract, keywords, funding, and other information from 7468 relevant publications. In addition, the applications and restrictions associated with the use of geopolymers in the construction sector are discussed, as well as possible solutions to overcome these restrictions. The scientometric analysis revealed that the leading publication source (journal) in terms of articles and citations is “Construction and building materials”; the mostly employed keywords are geopolymer, fly ash, and compressive strength; and the top active and contributing countries based on publications are China, India, and Australia. Because of the quantitative and graphical representation of participating nations and researchers, this study can help academics to create collaborative efforts and exchange creative ideas and approaches. In addition, this study concluded that the large-scale usage of geopolymer concrete is constrained by factors such as curing regime, activator solution scarcity and expense, efflorescence, and alkali–silica reaction. However, embracing the potential solutions outlined in this study might assist in boosting the building industry’s adoption of geopolymer concrete.

## 1. Introduction

Cement concrete is the most widely used material in construction [1,2,3,4], with annual worldwide consumption levels of over 30 billion tons [5,6,7], depleting significant amounts of ordinary Portland cement (OPC), the primary binder utilized to manufacture traditional concrete [8,9,10,11]. Nevertheless, the manufacture of OPC is linked to major environmental challenges, such as the intense consumption of natural resources and the generation of greenhouse gases [12,13,14,15]. Roughly 1.5 tons of raw ingredients are required to create a single ton of OPC, which generates approximately 0.55 tons of carbon dioxide directly, whilst the burning of fuels generates approximately 0.4 tons of carbon dioxide, for an overall of 0.8–1.0 tons of carbon-dioxide discharge [16]. The cement industry consumes a substantial quantity of fossil fuels, which accounts for around 12–15% of industrial energy [17,18,19,20]. Worldwide, it is projected that the production of OPC causes approximately 1350 million tons of greenhouse gases annually, which accounts for 6–9% of the world’s greenhouse-gas emissions [21]. This emission is primarily caused by burning fuel in the boiler, electrical-energy consumption, and decarbonation of limestone [22].

**Figure 1:**
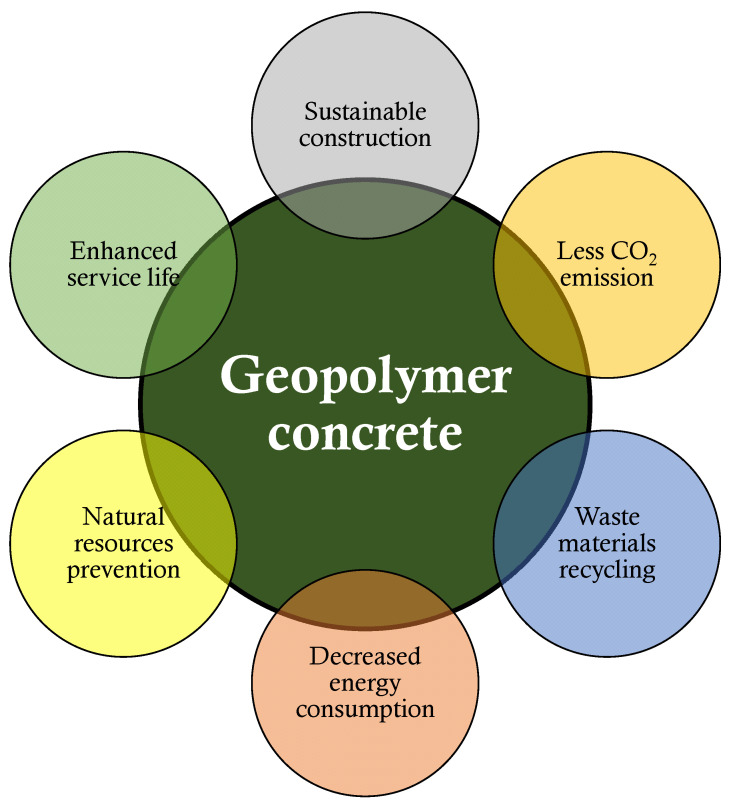
Advantages of geopolymer concrete made from waste materials [67].

Over the last three decades, the rising awareness of the ecological difficulties involved with the production of conventional concrete has prompted researchers and the building sector to seek for alternative techniques to generate eco-friendly materials [23,24,25,26]. The World Green Building Council (WGBC) 2019 issued the approaches and principles for designing eco-friendly structures, with a goal of reducing carbon footprints by 40% by 2030 and achieving zero carbon-dioxide emissions by 2050 [5]. Utilizing alternative fuels, absorbing carbon dioxide, increasing energy efficacy inside the boiler, substituting OPC with waste materials or nanoparticles, and manufacturing eco-friendly cementitious products and technologies are the primary steps advised. Utilizing alkali-activated materials [27,28], such as geopolymers [29], can be preferable to CCBC. The interaction between activators and precursors produces alkali-activated compounds. Based on the calcium content of the reaction products, they are divided into two classes: those rich in calcium, such as blast-furnace slag, with a Ca/(Si + Al) ratio larger than 1; and those low in calcium, such as geopolymers [27]. Consequently, geopolymers have emerged as the most effective substitutes for OPC [30,31,32,33,34,35,36]. In addition to the positive eco-friendly effect of geopolymers by decreasing consumption of energy and reusing waste materials, the improved durability and mechanical performance are accompanied by cost-effectiveness, as well as their exceptional resilience to elevated temperatures and acid attack [37,38,39,40,41,42,43,44]. Utilizing geopolymers as an alternative to OPC may also minimize the carbon-dioxide footprint when compared to conventional concrete [45,46,47,48,49,50]. As mentioned in prior studies, the rate of decreased emissions varies substantially, ranging from 9% [51] to 26–45% [52] to around 80% [53]. These enormous variances are mostly attributable to a few critical criteria, such as the convenience and closeness of raw ingredients, mix design, activator type/amount required and manufacturing process employed, and curing regime [5]. As geopolymer is a relatively new technology, it lacks design guidelines compared to conventional concrete; thus, further study and experimental testing are necessary to reduce the gaps in geopolymers applications [46]. Other issues restricting the uses of geopolymers include the demand for a high curing temperature requirement for several precursor materials, the high price of activator chemicals, the dearth of activator ingredients, efflorescence, and alkali–silica reaction (ASR) [23,54]. As geopolymers require raw materials with higher aluminosilicate concentrations that are present in waste materials, reusing these types of materials to produce geopolymers would reduce environmental pollution [55,56,57,58,59]. Figure 1 demonstrates that the application of these types of waste materials will benefit both the environment and the economy, because these waste materials are ample and the demand for affordable housing will increase as the population expands [60,61,62,63]. Globally, geopolymers are gaining popularity in the field of research, and have the possibility to become the most sustainable construction material [64,65,66].

As research on the geopolymers increases in response to growing environmental concerns associated with the use of ordinary cement concrete, scientists are confronted with information restrictions that may stifle innovative research and academic collaboration. Consequently, it is crucial to develop and implement a system that facilitates academics to acquire essential knowledge from as highly credible sources as possible. Using a software program, a scientometric technique may help overcome this deficiency. This project aims to conduct a scientometric study of bibliographic records published on geopolymers up to May 2022. A scientometric assessment can conduct a quantifiable assessment of an enormous amount of bibliographic data by utilizing the appropriate software application. Conventional review studies lack the capacity to accurately and comprehensively link disparate portions of the literature. Scientific visualization, co-citations, and co-occurrence are among the highly complicated aspects of contemporary research [11,47,67]. Scientometric analysis reveals the sources with the most publications, keyword co-occurrence, the authors with the most papers and citations, top articles in terms of citations, and the regions actively involved in the research of geopolymers. The Scopus search engine was used to obtain containing citations, bibliographic, abstract, keywords, funding, and other information from 7468 pertinent papers, which were then analyzed employing the VOSviewer application. As a result of the graphical and statistical representation of researchers and nations, this study will assist scholars in developing collaborative endeavors and exchanging innovative concepts and techniques. Following a scientometric analysis of subject-related keywords and a review of the most pertinent literature, this study highlighted and discussed the current-state applications of geopolymer concrete, the limitations associated with the production and use of geopolymer composites, and potential solutions.

## 2. Review Strategy

**Figure 2:**
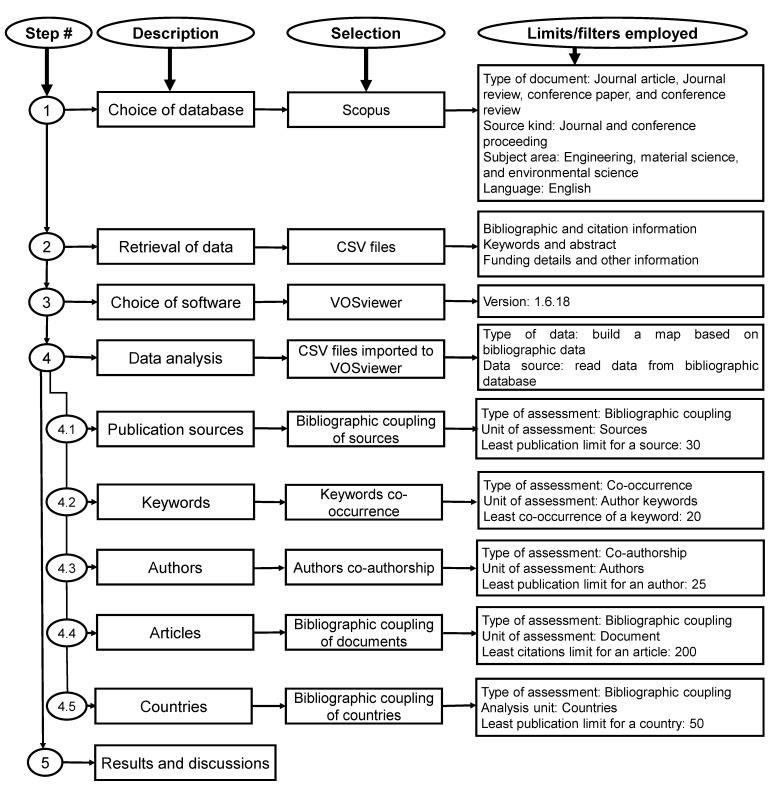
Sequence of the review’s approach signifying various options selected and limits applied.

This study conducted a scientometric analysis of bibliographic data [68,69,70] in order to quantify the numerous characteristics of bibliographic data. Scientometric studies utilize scientific mapping, a technique established by academics for bibliometric data analysis [9,71]. Numerous articles have been recorded on the subject; thus, it is essential to utilize a credible search engine. Web of Science and Scopus are two extremely precise databases that are ideally fit for this objective [72,73]. Scopus, which comes highly recommended by academics, was used to collect bibliographic information for this study on geopolymers. As of May 2022, a Scopus search for the term “geopolymers” yielded 10,115 results. Numerous filter settings were utilized to eliminate unnecessary papers. Figure 2 depicts a complete flowchart of the data retrieval, analysis, and numerous limits/filters applied during the analysis. Additionally, other studies have reported on the same method [74,75,76]. Following the application of these filters to the Scopus database, 7468 results remained. Scopus records were stored in a Comma Separated Values (CSV) format for further assessment using the relevant software. VOSviewer (version 1.6.18) was utilized to construct the scientific visualization and quantitative evaluation of the obtained material. VOSviewer is a freely accessible, open-source mapping tool that is generally employed in distinct study areas and proposed by academics [77,78,79]. Consequently, the current study’s objectives were met by the usage of VOSviewer. The resulting CSV file was loaded into the VOSviewer, and further evaluation was conducted while maintaining data consistency and reliability. During the scientometric analysis, the publishing outlets, the most frequently occurring keywords, the researchers with the highest number of published articles and citations, documents that received the most citations, and the state’s involvement were evaluated. The multiple features, their interrelationships, and co-occurrences were illustrated via maps, and their quantitative data were presented in tables. The color of an item in a map is determined by the cluster to which the item belongs. In addition, for the density visualization, there are various color options such as viridis, plasma, and rainbow, and this study used the rainbow option for density mapping.

## 3. Results and Discussions

### 3.1. Subject Area and Yearly Publication of Documents

**Figure 3:**
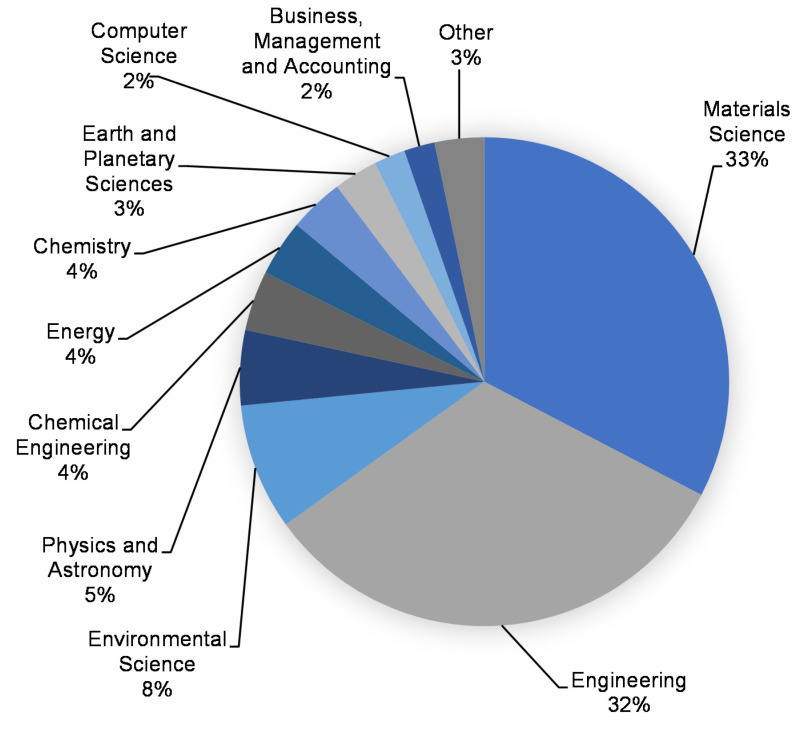
Subject area of documents in the research of geopolymers.

**Figure 4:**
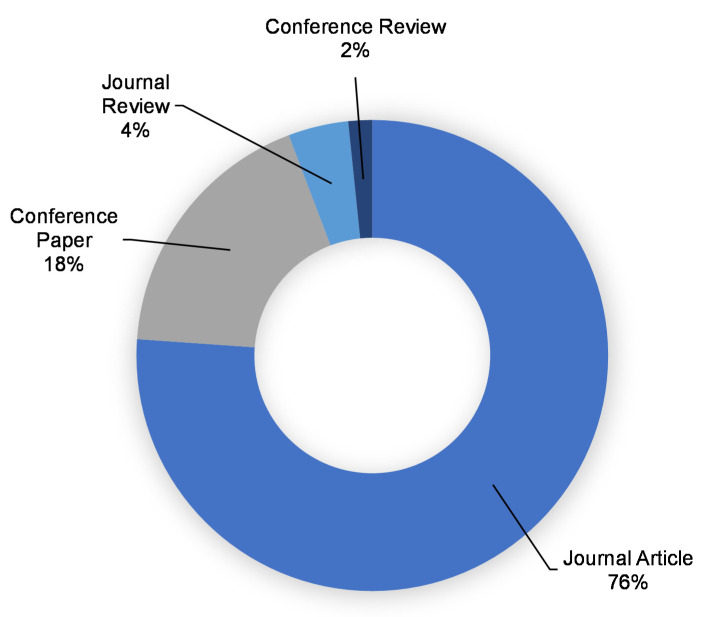
Kind of documents published on the research of geopolymers.

**Figure 5:**
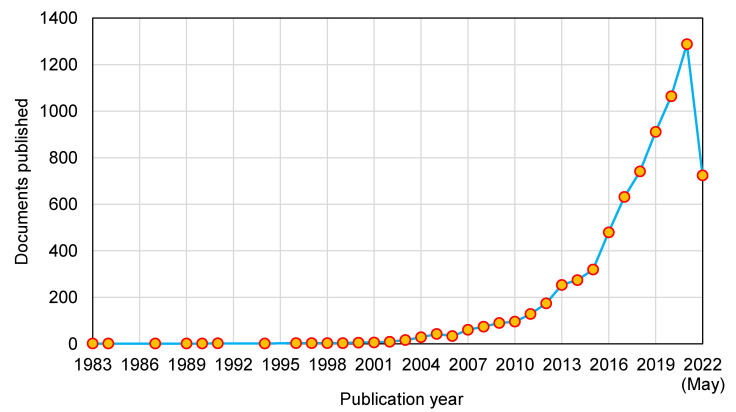
Publication year of documents on the research of geopolymers up to May 2022.

This assessment was performed using the Scopus analyzer to identify the most pertinent study fields. As seen in Figure 3, Materials Science, Engineering, and Environmental Science were determined to be the top three document-generating disciplines, with about 33%, 32%, and 8% of documents, respectively, contributing a total of 73% of documents. In addition, the Scopus database was analyzed for the kind of publications containing the documents on the subject research area (Figure 4). Based on this assessment, journal papers, conference articles, journal reviews, and conference reviews comprised around 76%, 18%, 4%, and 2% of all data, respectively. Figure 5 depicts the annual development of articles published in the current study field from 1983 to May 2022, since the first document on the subject research field was found in 1983. Up until 2000, there was almost a negligible growth in the publications in the field of geopolymers research, with an average of around two papers each year. Following that, there was a modest increase in the number of articles, with an average of around 91 articles each year between 2001 and 2015, with 319 articles in 2015. The number of publications increased significantly from 2016 onwards, with an average of 852 publications between 2016 and 2021, with 1288 publications in 2021. The quantity of publications is increasing each year, and in the current year, the number of publications on the subject research area is 724 so far (May 2022). It is fascinating to see that the researchers are focusing their attention on the use of alternative binders for construction materials.

### 3.2. Bibliographic Coupling of Publication Sources

**Figure 6:**
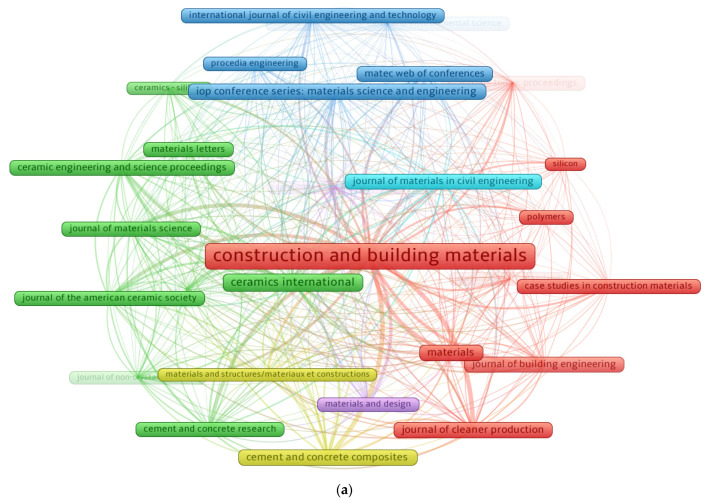

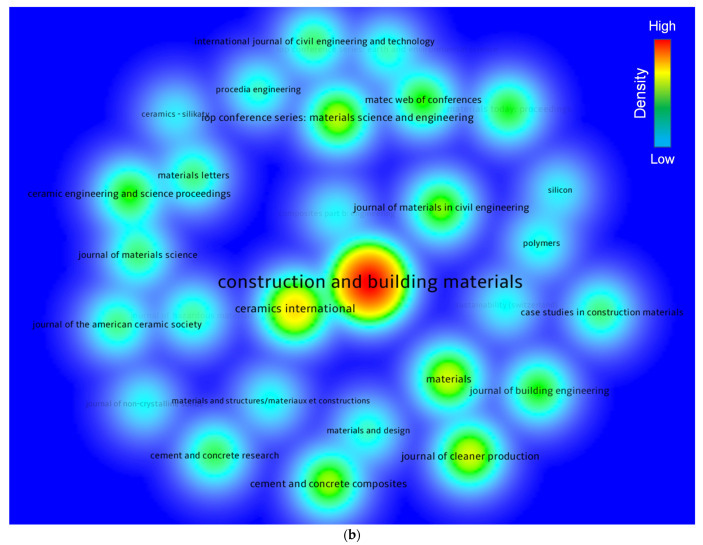
Sources mapping with a minimum of 30 publications. (**a**) Network visualization; (**b**) density.

**Figure 7:**
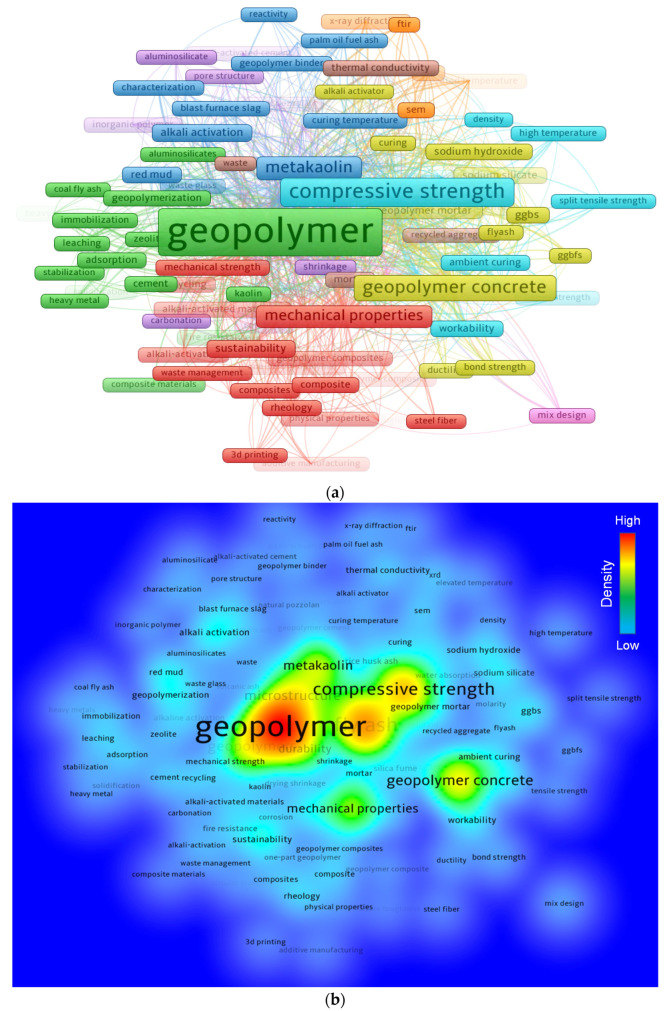
Keywords co-occurrence. (**a**) Scientific mapping; (**b**) density.

The evaluation of publication outlets (journals) was performed on the bibliographic data employing the VOSviewer tool. A minimum of 30 papers per source was stipulated, and 28 of the 753 sources satisfied this requirement. Table 1 displays the publishing outlets that released at least 30 publications on geopolymers research up to May 2022, along with the number of citations received within that time frame. “Construction and building materials (CONBUILDMAT)”, “Ceramics international”, and “Journal of cleaner production” were found to be the top publication journals with 770, 221, and 151 papers, respectively. Furthermore, the top three sources based on the number of citations received up to May 2022 are “CONBUILDMAT” with 34,289; “Cement and concrete research” receiving 12,256; and “Journal of materials science” with 9547 citations. Exceptionally, this examination would provide a groundwork for forthcoming scientometric evaluations in the research for geopolymers. Additionally, previous conventional review studies were unable of producing systematic graphs. Figure 6 depicts a visualization of sources publishing at least 30 articles. The frame dimension in Figure 6a is related to the outlet’s influence on the present study field based on document count; a bigger frame size indicates a greater impact. As an illustration, “CONBUILDMAT” has a larger frame than the others, indicating that it is a journal of great significance in the present research field. Six groups/clusters were formed, characterized by a distinct color on the map (blue, red, purple, yellow, cyan, and green). Groups/clusters are developed based on the extent of the research outlet or the frequency with which they are co-cited in comparable articles [80]. The VOSviewer grouped journals according to their co-citation tendencies in published articles. For example, the red cluster comprises nine journals that have been co-cited many times in the same work. In addition, the links between closely located frames (sources) in a group/cluster are greater than those between widely spread. For example, “CONBUIDMAT” correlates more strongly with “Case studies in construction materials” than with “Materials” or “Journal of building engineering”. As seen in Figure 7b, various shades correspond to varying density concentrations for a journal. Red has the highest density concentration, followed by yellow, green, and blue. “CONBUILDMAT” has a red shade implying its higher contribution to the research of geopolymers.

### 3.3. Co-Occurrence of Keywords

Keywords are significant in research since they distinguish and emphasize the basic subject of the study domain [81]. The minimum repetition requirement for a keyword was kept at 20, and 108 of the 7727 keywords were preserved. Table 2 records the leading 30 keywords most frequently used in published works on the subject. The five most often-occurring terms in the topic study field are geopolymer, fly ash, compressive strength, geopolymer concrete, and geopolymers. According to the keyword analysis, geopolymer has mostly been investigated to manufacture a sustainable construction material and is mostly researched to be produced from waste materials, such as fly ash, slag, silica fume, rice husk ash, etc. Figure 7 illustrates a systematic graph of keywords based on co-occurrences, connections, and density proportional to their occurrence frequency. A keyword’s frame size in Figure 7a signifies its frequency, while its position suggests its co-occurrence in articles. In addition, the graph expresses that the top keywords have larger frames than the rest, signifying that these are essential keywords for a real investigation in the research of geopolymers. The graph highlights clusters in a manner that shows their co-occurrence in a variety of published documents. The color-encoded grouping is determined by the co-occurrence of several keywords in publications. Nine clusters are represented by diverse shades in Figure 7a. As observed in Figure 7b, distinct colors represent differing keyword density concentrations. The shades red, yellow, green, and blue are arranged according to their density strengths, with red representing the highest density concentration and blue representing the lowest. Geopolymer, fly ash, compressive strength, and other prominent keywords display red or yellow signals indicating a greater density of occurrences. This finding will help ambitious researchers select keywords that ease the discovery of published papers on a specific topic.

### 3.4. Authors’ Coauthorship

**Figure 8:**
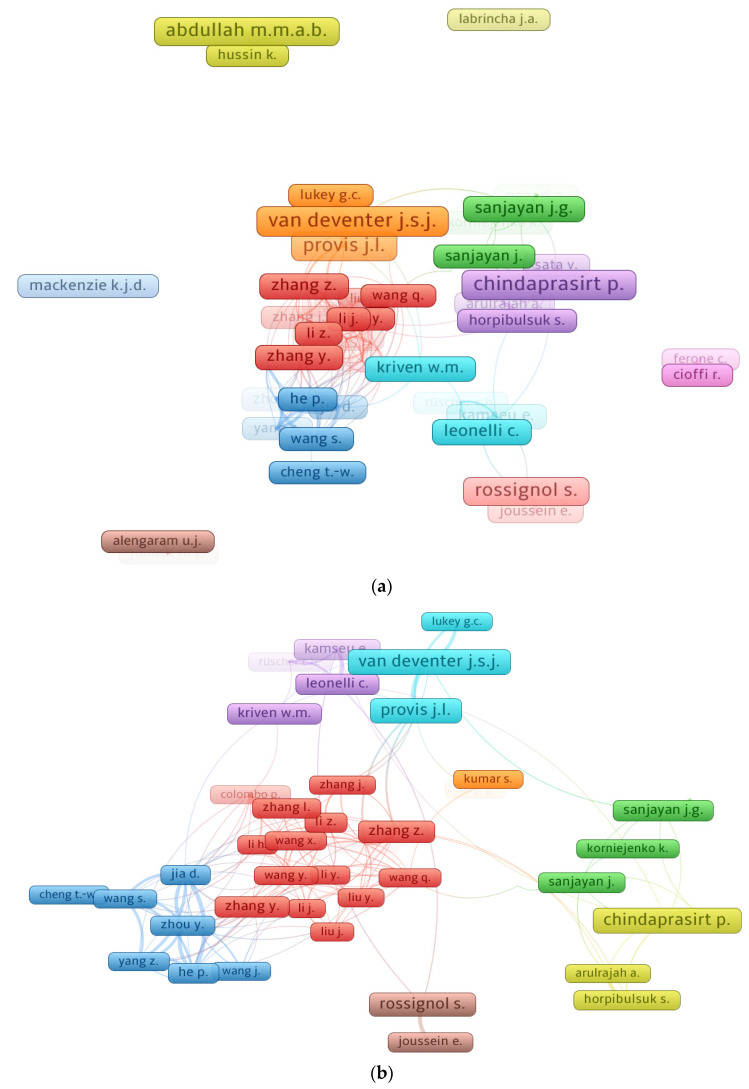
Science mapping of authors. (**a**) Authors with a least 25 publications; (**b**) connected authors based on citations.

Citations show a scientist’s impact in a particular field of research [82]. The lowest paper threshold for a researcher was decided at 25, and 55 out of 9855 researchers satisfied this requirement. The authors with the most articles and citations in the field of geopolymers, as assessed from the bibliographic data using VOSviewer, are included in Table 3. Each author’s average citations were determined by dividing the total citations by the total number of articles. It is complicated to assess the effectiveness of a scientist when all parameters, such as the quantity of documents, overall citations, and average citations, are taken into account. Alternatively, the researcher’s ranking will be evaluated separately for each component, i.e., the number of documents, the number of overall citations, and the average number of citations. The analysis revealed that Van Deventer J.S.J. and Chindaprasirt P. are the most prolific researchers with 93 publications each, followed by Provis J.L. with 86 and Abdullah M.M.A.B. with 79 publications. In terms of total citations, Van Deventer J.S.J. leads the field with 18,335, Provis J.L. is second with 15,257, and Lukey G.C. is third with 9891 overall citations in the present research domain. In addition, when the average number of citations is compared, the authors might be ranked as Lukey G.C. at the top with nearly 341 average citations, Van Deventer J.S.J. at second with about 197, and Provis J.L. is third with approximately 177 average citations. Figure 8 depicts the association between writers with at least 25 publications and the most notable authors. Figure 8a depicts the scientific mapping of scholars who have contributed at least 25 papers to the current field of study. Figure 8b depicts the largest group of related writers based on citations, which consists of 44 of the 55 authors. This investigation indicated that the majority of researchers working on geopolymers are linked via citations.

### 3.5. Bibliographic Coupling of Documents

**Figure 9:**
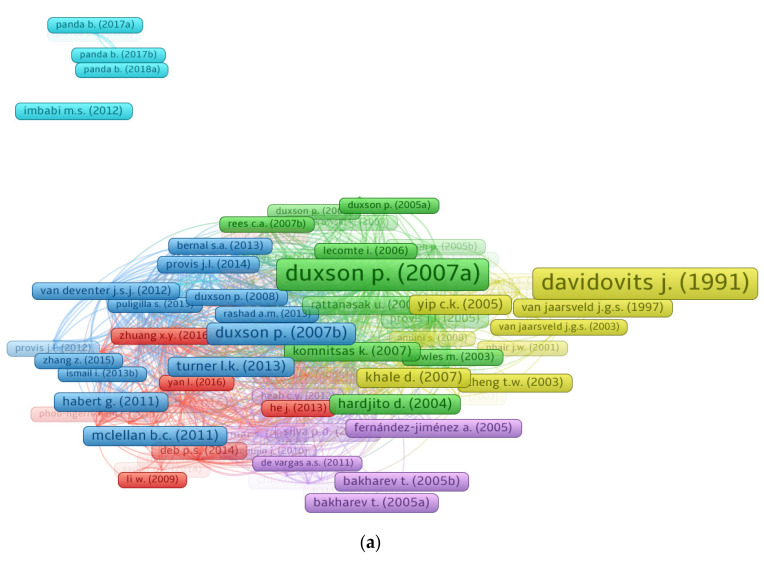

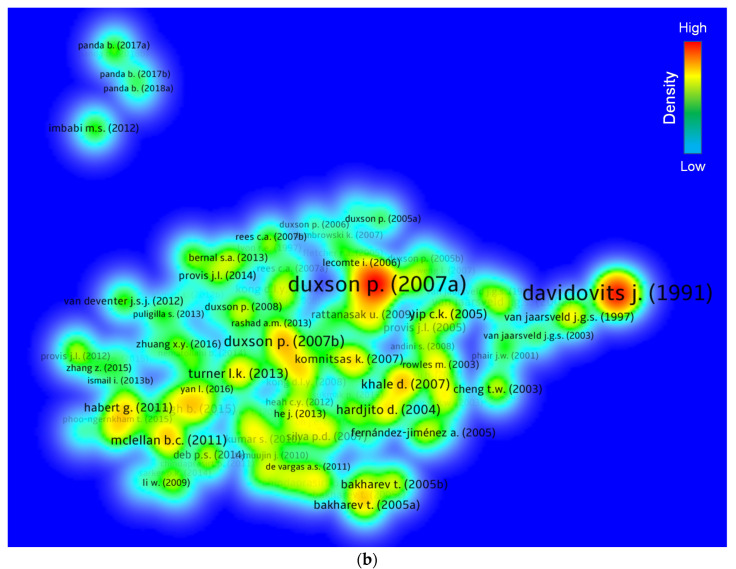
Systematic map of published documents. (**a**) Linked documents based on citations; (**b**) connected documents density.

The number of citations a publication receives signifies its impact in a certain research domain. In their respective study domains, papers having a large number of citations are regarded as pioneering. The lowest number of citations for a document was kept at 200, and 131 out of 7468 papers met this threshold. In Table 4, the top five articles in the field of geopolymers based on citations are included, along with their authors and citation counts. The work titled “Geopolymer technology: The current state of the art” by Duxson P. [53] has 2573 citations. Davidovits J. [83] and Duxson P. [84] received 2507 and 1124 citations for their articles, respectively, and were placed in the top three. However, until May 2022, just 28 papers had acquired more than 500 citations. Moreover, Figure 9 depicts the scientific visualization of connected articles on the basis of citations and the density concentration of these articles in the domain of the present study. Figure 9a displays that 128 of 131 publications were linked by citations, as determined by the VOSviewer analysis. In addition, the density mapping (Figure 9b) demonstrates the increased density concentration of the top articles based on citations.

### 3.6. Bibliographic Coupling of Countries

**Figure 10:**
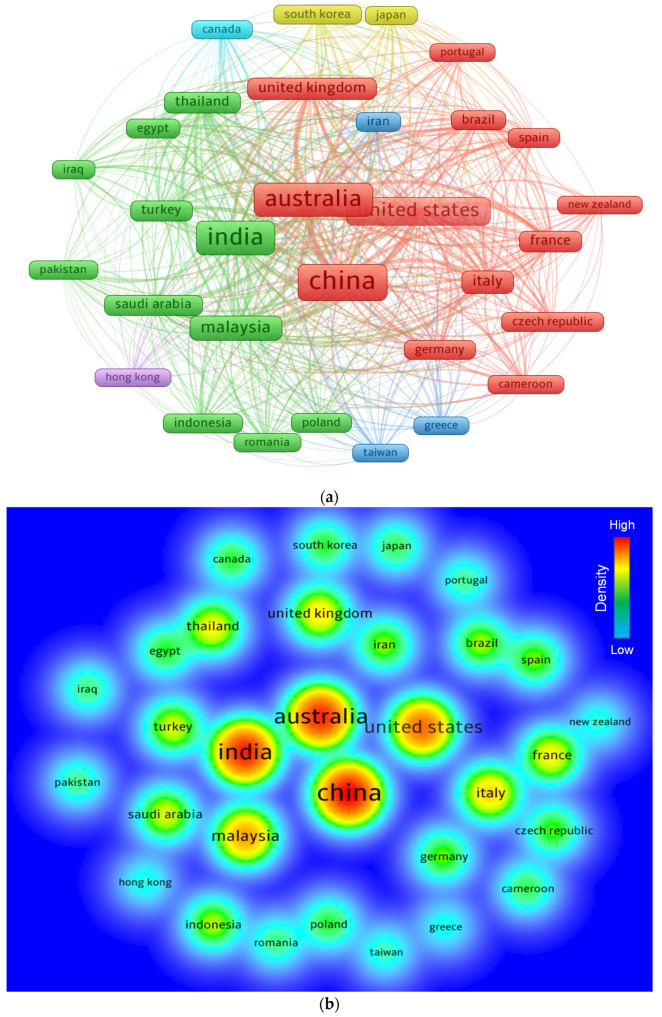
Science mapping of countries with minimum of 50 publications. (**a**) Network map; (**b**) density.

Numerous nations have presented more documents to the present research area than others, and they plan to continue their contributions. The systematic map was constructed so that readers may examine regions devoted to geopolymers research for predicting concrete properties. The minimum number of documents a nation may possess was kept 50, and 31 nations satisfied this threshold. The countries included in Table 5 have produced a minimum of 50 documents on the current topic of research. China, India, and Australia gave the greatest number of papers, with 895, 776, and 743 documents, respectively. In addition, Australia received 54,555 citations, followed by China with 22,820 citations, and the United States received 15,649 citations. Figure 10 depicts the systematic map and the density strength of countries linked by citations. Figure 10a depicts that the size of a frame is proportional to a country’s impact on the topic study based on the number of articles. As seen in Figure 10b, the nations with the greatest level of participation had a greater density. The graphical depiction and quantitative record of the participating nations will assist young scientists in making scientific alliances, launching collaborative ventures, and exchanging creative methods and concepts. Scholars from states concerned with developing research on geopolymers can collaborate with experts in the field and profit from their expertise.

## 4. Discussion

This systematic review performed the statistical analysis and mapping of the bibliographic data available in the research of geopolymers. Previous manual review studies lacked the capacity to completely and precisely link disparate areas of the literature. This analysis identified the sources of publications (journals) that published the most documents, the most often-used keywords in publications, the documents and researchers with the highest citations, and the nations vigorously engaged in geopolymers research. According to the assessment of keywords, geopolymer has mostly been researched to produce sustainable construction material and is mostly investigated to be produced from waste materials such as fly ash, slag, silica fume, rice husk ash, etc. In addition, the literature and their linkages based on citations were used to identify the highly committed and participating nations based on publication count. The graphical representation and quantitative analysis of the participating countries and researchers will help young scientists in forming scientific partnerships, establishing joint ventures, and sharing advanced methods and concepts. Scholars from countries concerned with expanding the research on the application of geopolymers can collaborate with professionals in the discipline and benefit from their expertise. After assessing keywords in the subject topic using the scientometric analysis method and reviewing the most relevant literature, this study highlighted and discussed the present-state applications of geopolymer concrete, and the limitations associated with the production and use of geopolymer composites and possible solutions in the following subsections.

### 4.1. Applications of Geopolymer Concrete

Geopolymer technology has a significant possibility for reusing waste materials, thus solving the problem of environmental pollution caused by the disposal of these items in landfills. In the domains of fire-resilient and asbestos-free materials, high-tech materials, new ceramics, and harmful waste-stabilization matrices, geopolymer-based products have been utilized [85]. Cement and concrete are essential to modern development because they facilitate the creation of the infrastructure required for a superior level of livelihood. It is believed that geopolymers provide a road for more commercialization, large-scale production, and a basis for rapid interpretation of research pertaining to comments regarding the eco-responsiveness of building materials. In addition, an evaluation of the optimal composition and transformation conditions must be conducted with respect to the geography and supply chains of raw materials [86].

The use of geopolymer concrete in various structures such as precast bridge decks, retaining walls, road pavements, aircraft pavements, water tanks, boundary blocks, and precast beams has increased in Australia [87]. Other studies have established the use of geopolymer concrete as a structural element, justifying its current usage in Australia and parts of Europe. Recognition and implementation of geopolymer concrete are achieving traction. In 2016, the government of Japan established a committee to study the current usage of geopolymer concrete structural elements in different global locations. The International Union of Laboratories and Experts in Construction Materials, Systems, and Structures (RILEM, after its French acronym) has tasked a committee with recognizing and validating methods for evaluating the durability of geopolymer composites. The study comprised 15 laboratories from around the world. Since geopolymer composites do not have a lengthy track record of durability [88,89], it is crucial to establish these test and validation procedures. In particular places worldwide, the application of geopolymer concrete is increasing quickly, whilst in others, it is advancing gradually [90]. Alkali-activated binders might be a helpful ingredient of eco-friendly construction materials if they are created effectively, their carbon impact is considered, and local resources are utilized. Because of the necessity for exact mix design and curing, technical issues connected with certain kinds of applications, and supply chain restrictions, it is doubtful that geopolymer concrete will be able to replace conventional concrete in a like-for-like manner. Furthermore, even if regulatory authorities understood the results of durability experiments on geopolymer concrete, the procurement of raw ingredients for its production would be a barrier. In addition, since the supply chain for cement-based composites depends on OPC, controlling and product-assurance obstacles must be overwhelmed to permit the usage and approval of geopolymer composites. Moreover, it is essential to emphasize that users, academia, and regulatory bodies recognize the commercialization potential of geopolymer composites [91,92]. Manufacturing geopolymer tiles for increased temperatures is another possible application of geopolymer composites [93,94,95].

### 4.2. Limitations and Potential Remedies

Since geopolymers eliminate the need for OPC in building materials, the introduction of geopolymer composites has created new opportunities for sustainability in the building industry. However, there are a number of constraints that limit their future applicability.

- Geopolymer composites need steam/heat curing for strength enhancement [96,97], which is challenging to apply to structural elements on-site. Nevertheless, enclosing the structural elements with films and creating a humid atmosphere might be an alternate curing method for geopolymer composites [98,99].
- Sodium-silicate deficiency is a further significant factor limiting geopolymer production. The environmental efficacy and financial advantage of geopolymers are highly dependent on the amount of alkali-activated material employed [23]. Geopolymer composites are also costly because of the high cost of activating solutions [100]. Utilizing rice husk ash as a silicon source in the production of sodium-silicate solution might reduce the requirement for sodium carbonate and quartz sand [101], both of which produce greenhouse gases during the production. Sodium silicate produced from rice husk ash and waste glass is an outstanding activator for the production of metakaolin-based geopolymers [102]. Utilizing glass-polishing waste as an activator may also be a cost-efficient option [103].
- Another factor that impacts the advancement of geopolymer composites is efflorescence. Salt growth in surface alkali-activated cement has been seen in several industrial applications and laboratory experiments, where it has been described as efflorescence. In silicon-rich settings, high concentrations of alkali activators can induce substantial efflorescence. The level of efflorescence decreased as the content of alumina increased [104]. The occurrence of geopolymer efflorescence decreases as silica concentration and grain size increase [105,106]. The use of nanosilica in geopolymer composites based on metakaolin is beneficial. Nanosilica decreased efflorescence by consuming alkali ions in excess from the pore solution, therefore forming an amorphous gel phase [107].
- The use of alkaline activators in greater concentrations may hasten ASR, hence restricting the usefulness of geopolymer composites. According to the researchers, ASR expansion was more easily generated in high-calcium and mixed systems than in low-calcium alkali-activated cement systems. Activators of varying kinds and dilutions can also induce ASR. Certain admixtures may lead to the development of ASR [108]. Therefore, further study on ASR is necessary to determine the crucial factors that influence its incidence.

## 5. Conclusions

This study’s purpose was to undertake a scientometric assessment of the available literature on geopolymers to evaluate different metrics. The database Scopus was searched for 7468 related articles, and the records were evaluated employing the VOSviewer application. The following conclusions were obtained from this study:

- An evaluation of publication journals, including articles on geopolymers research, revealed that “CONBUILDMAT”, “Ceramics international”, and “Journal of cleaner production” are the top three sources, with 770, 221, and 151 publications, respectively. In terms of total citations, the top three publishing sources are “CONBULDMAT” with 34,289, “Cement and concrete research” with 12,256, and “Journal of materials science” with 9547 citations.
- Assessment of keywords on the topic research field reveals that geopolymer, fly ash, compressive strength, geopolymer concrete, and geopolymers are the five most often occurring terms. The keyword analysis found that geopolymer has mostly been researched to produce sustainable construction material and is mostly investigated to be produced from waste materials, such as fly ash, slag, silica fume, rice husk ash, etc.
- Analysis of researchers found that just 55 authors had published at least 25 articles on geopolymers research. According to their document count, overall citations, and average citations, the leading authors were categorized. Van Deventer J.S.J. and Chindaprasirt P. are the most prolific researchers with 93 publications each, followed by Provis J.L. with 86 and Abdullah M.M.A.B. with 79 publications. In terms of total citations, Van Deventer J.S.J. leads the field with 18,335, Provis J.L. is second with 15,257, and Lukey G.C. is third with 9891 overall citations in the present research domain. In addition, when the average number of citations is compared, the authors might be ranked as Lukey G.C. at the top with nearly 341 average citations, Van Deventer J.S.J. at second with about 197, and Provis J.L. in third with approximately 177 average citations.
- An assessment of published documents containing data on geopolymers revealed that Prasanna P. [53] ’s work “Geopolymer technology: The current state of the art” received 2573 citations. Davidovits J. [83] and Duxson P. [84] received 2507 and 1124 citations for their studies, respectively, and were among the top three. In addition, as of May 2022, just 28 papers had received more than 500 citations in the topic field.
- Based on their engagement in geopolymers research, the top countries were evaluated, and it was concluded that only 31 countries published at least 50 documents. The United States, China, and Indi China, India, and Australia presented 895, 776, and 743 documents, respectively. In addition, Australia received 54,555 citations, followed by China with 22,820 citations, and the United States received 15,649 citations.
- Since geopolymers require source materials with greater aluminosilicate concentrations, which are prevalent in various waste materials such as fly ash, slag, rice husk ash, etc., recycling these materials to create geopolymers would minimize environmental pollution.
- The large-scale applications of geopolymer concrete in the building sector are limited due to several restraints such as curing regime, deficiency and cost of activator solution, efflorescence, and ASR. Adopting potential remedies as discussed in this study might help increase the acceptance of geopolymer concrete in construction. However, further in-depth investigations are necessary on these solutions for the large-scale applicability of geopolymers.

## Figures and Tables

**Table 1:** List of publication outlets with at least 30 publications in the research of geopolymers up to May 2022.

S/N	Source of Publication	Publication Count	Total Citations Received
1	Construction and building materials	770	34,289
2	Ceramics international	221	7430
3	Journal of cleaner production	151	7772
4	Materials	148	1807
5	IOP conference series: Materials science and engineering	135	633
6	Cement and concrete composites	124	8314
7	Journal of materials in civil engineering	114	3083
8	Ceramic engineering and science proceedings	100	742
9	MATEC web of conferences	99	591
10	Journal of building engineering	98	1048
11	Materials today: Proceedings	94	374
12	Cement and concrete research	81	12,256
13	International journal of civil engineering and technology	73	352
14	Journal of materials science	72	9547
15	Materials letters	70	2537
16	Case studies in construction materials	70	532
17	Journal of the American ceramic society	68	3387
18	Journal of hazardous materials	61	5762
19	IOP conference series: earth and environmental science	53	54
20	Materials and design	52	4834
21	Procedia engineering	51	1713
22	Polymers	46	176
23	Materials and structures/materiaux et constructions	43	2327
24	Sustainability (Switzerland)	41	279
25	Journal of noncrystalline solids	39	1978
26	Composites part b: Engineering	38	2680
27	Silicon	36	113
28	Ceramics-Silikaty	34	1161

**Table 2:** List of to 30 most used keywords in the research of geopolymers.

S/N	Keyword	Occurrences
1	Geopolymer	2064
2	Fly ash	953
3	Compressive strength	692
4	Geopolymer concrete	484
5	Geopolymers	401
6	Microstructure	395
7	Metakaolin	348
8	Mechanical properties	311
9	Durability	209
10	Strength	142
11	Slag	132
12	Sustainability	106
13	Alkali activation	97
14	Porosity	93
15	Concrete	91
16	Workability	83
17	Flexural strength	81
18	GGBS	77
19	Geopolymer mortar	76
20	Red mud	75
21	Rice husk ash	75
22	Sodium silicate	75
23	Sodium hydroxide	66
24	Silica fume	65
25	Ground granulated blast furnace slag	64
26	Geopolymerization	62
27	Rheology	61
28	Ambient curing	58
29	Thermal conductivity	57
30	SEM	54

**Table 3:** List of scholars having at least 25 publications in the research of geopolymers up to May 2022.

S/N	Author Name	Articles Published	Total Citations Received	Average Citation Count
1	Van Deventer J.S.J.	93	18,335	197
2	Chindaprasirt P.	93	7598	82
3	Provis J.L.	86	15,257	177
4	Abdullah M.M.A.B.	79	857	11
5	Rossignol S.	78	1790	23
6	Zhang Z.	61	2760	45
7	Sanjayan J.G.	56	3577	64
8	Leonelli C.	56	1688	30
9	Kriven W.M.	52	1439	28
10	Zhang Y.	52	885	17
11	Kamseu E.	51	1296	25
12	Sanjayan J.	47	2231	47
13	He P.	46	1391	30
14	Wang H.	44	2668	61
15	Jia D.	43	1392	32
16	Mackenzie K.J.D.	42	2725	65
17	Nazari A.	41	1523	37
18	Zhang L.	39	1546	40
19	Sandu A.V.	39	503	13
20	Zhou Y.	38	1134	30
21	Li Z.	38	852	22
22	Horpibulsuk S.	37	2293	62
23	Joussein E.	37	994	27
24	Shaikh F.U.A.	36	2162	60
25	Arulrajah A.	36	1856	52
26	Wang S.	36	911	25
27	Li J.	35	262	7
28	Kumar S.	33	1838	56
29	Wang Y.	33	398	12
30	Hussin K.	32	515	16
31	Korniejenko K.	32	318	10
32	Van Riessen A.	31	3829	124
33	Sata V.	31	2670	86
34	Zhang J.	31	1487	48
35	Yang Z.	31	517	17
36	Li Y.	30	431	14
37	Lukey G.C.	29	9891	341
38	Kamarudin H.	29	1518	52
39	Cioffi R.	29	1500	52
40	Wang Q.	29	227	8
41	Sarker P.K.	28	2774	99
42	Cheng T.-W.	28	591	21
43	Liu Y.	28	348	12
44	Alengaram U.J.	27	1928	71
45	Castel A.	27	1197	44
46	Ferone C.	27	1132	42
47	Colombo P.	27	986	37
48	Wang J.	27	263	10
49	Labrincha J.A.	26	1222	47
50	Rüscher C.H.	26	444	17
51	Dai J.-G.	26	422	16
52	Wang X.	26	199	8
53	Liu J.	26	130	5
54	Jumaat M.Z.	25	2055	82
55	Li H.	25	369	15

**Table 4:** List of top 5 articles in terms of citations received up to May 2022.

S/N	Article	Title	Citations Received
1	Duxson P. [53]	Geopolymer technology: The current state of the art	2573
2	Davidovits J. [83]	Geopolymers-Inorganic polymeric new materials	2507
3	Duxson P. [84]	The role of inorganic polymer technology in the development of ‘green concrete’	1124
4	Mclellan B.C. [52]	Costs and carbon emissions for geopolymer pastes in comparison to ordinary portland cement	870
5	Turner L.K. [51]	Carbon dioxide equivalent (CO_2_-e) emissions: A comparison between geopolymer and OPC cement concrete	862

**Table 5:** List of countries that have presented at least 50 papers in the subject research domain up to May 2022.

S/N	Country	Documents Published	Overall Citations
1	China	895	22,820
2	India	776	12,041
3	Australia	743	54,555
4	United States	516	15,649
5	Malaysia	364	10,334
6	Italy	265	7953
7	United Kingdom	231	10,077
8	France	209	8825
9	Thailand	205	10,254
10	Saudi Arabia	179	3391
11	Turkey	178	3011
12	Indonesia	150	1355
13	Brazil	144	2718
14	Iran	136	3529
15	Spain	130	7315
16	Germany	125	3405
17	Czech Republic	120	3108
18	Egypt	107	2421
19	South Korea	106	2497
20	Poland	105	1012
21	Canada	104	2101
22	Cameroon	96	2893
23	Japan	94	3038
24	Iraq	81	1078
25	Romania	80	1121
26	Pakistan	77	903
27	Portugal	75	2460
28	Taiwan	64	1630
29	New Zealand	56	3042
30	Hong Kong	54	1466
31	Greece	52	2748

## Data Availability

The data used in this research have been properly cited and reported in the main text.
